# Effects of Dietary Fiber Fermentation and Protein Digestion Properties on Growth Performance and Microbial Metabolites in Weaned Pigs

**DOI:** 10.3390/ani15111669

**Published:** 2025-06-05

**Authors:** Jingyi Huang, Zhiqiang Sun, Qi Zhu, Fudong Zhang, Changhua Lai, Jinbiao Zhao

**Affiliations:** State Key Laboratory of Animal Nutrition and Feeding, College of Animal Science and Technology, China Agricultural University, Beijing 100193, China; 13370102152@163.com (J.H.); sunbeibei5421@163.com (Z.S.); s20243040844@cau.edu.cn (Q.Z.); zhangfd@cau.edu.cn (F.Z.); laichanghua999@163.com (C.L.)

**Keywords:** fiber fermentation speed, protein digestion speed, weaned pigs, short-chain fatty acids, microbial composition

## Abstract

Dietary nutrient digestion and utilization patterns influence pig performance and intestinal health. This study aimed to evaluate the effects of protein digestion and fiber fermentation speed among different feed ingredients on growth performance and fecal short-chain fatty acid (SCFA) concentrations in weaned pigs. A total of 192 weaned pigs (Duroc × Landrace × Yorkshire [6.87 ± 0.14 kg]) were selected and randomly divided into four dietary groups: fast-digesting protein with fast-fermenting fiber, fast-digesting protein with slow-fermenting fiber, slow-digesting protein with fast-fermenting fiber, and slow-digesting protein with slow-fermenting fiber. The results showed that cottonseed and wheat protein powders exhibited faster protein digestion than potato protein powder (*p* < 0.05). In vitro microbial fermentation of hawthorn powder and orange pomace resulted in greater and faster gas production and SCFA concentrations than sugarcane bagasse (*p* < 0.05). Orange pomace increased the abundance of *Klebsiella* and *Escherichia–Shigella*, whereas sugarcane bagasse increased the abundance of *Rikenellaceae_RC9_gut_group* and *norank_f__Muribaculaceae*. In addition, the fast-fermentation fiber tended to increase the daily weight gain and feed intake of piglets (*p* < 0.10), and the slow-fermentation fiber significantly reduced diarrhea incidence in pigs (*p* < 0.05). Fast fermentation increased acetate and valerate concentrations, and slow-digestion protein increased branched-chain SCFA and valerate contents (*p* < 0.05). In conclusion, there were large variations in protein digestion and fiber fermentation speed among the different common feed ingredients. Dietary protein digestion and fiber fermentation speed would affect growth performance and diarrhea incidence in weaned pigs.

## Simple Summary

The digestibility coefficients of dietary protein and fiber present in different ingredients used in feed for weaned pigs have been widely studied. It is important to provide practical insights into optimizing dietary formulations to enhance the gut health and growth performance of pigs during the weaning period, based on protein digestion and fiber fermentation speed. The findings of our study showed that the digestion speed of dietary protein did not affect the growth performance of weaned pigs. However, rapid fiber fermentation tended to increase average daily gain, whereas slow fiber fermentation reduced the incidence of diarrhea in weaned pigs.

## 1. Introduction

The weaning period represents a critical developmental stage for piglets, characterized by significant physiological and environmental challenges. During this phase, piglets often experience reduced growth performance, nutrient digestion disturbances, and increased susceptibility to infectious diseases due to the immaturity of their gastrointestinal tract and immune system [1]. Dietary nutrient composition plays a crucial role in supporting gut health and optimizing growth performance in weaned pigs [2]. For example, highly digestible protein in the diet can promote nutrient absorption, while fermentable fibers are degraded by the gut microbiota to produce short-chain fatty acids (SCFAs), which play a vital role in maintaining intestinal barrier function and optimizing the balance of the gut microbiota [3,4].

The effects of high-protein and high-fiber feed ingredients on the growth performance and intestinal health of weaned pigs have been studied based on the protein digestibility and microbial fermentability of fiber components [5]. The interactions between dietary protein and fiber levels on the growth performance, nutrient digestibility, and intestinal microbial community of weaned pigs have also been explored [6]. However, the digestibility coefficients of dietary protein and fiber only reflect the amount of nutrients that can be digested by endogenous enzymes; they cannot reflect the digestion and fermentation kinetics processes of dietary protein and fiber in the pig intestine [7]. Many previous studies have reported that the nutrient digestion speed of feed ingredients affects the energy-supply efficiency of starch and the conversion and deposition efficiency of protein [8]. The starch digestion characteristics of different cereal sources have been widely studied [9,10]. A recent study reported that different starch digestion speeds could regulate the kinetics pattern of dietary glucose release, and the corn–barley diet exhibited a better glucose release kinetic pattern than the corn–sorghum and corn–cassava diets, which could affect the portal amino acid contents and patterns by regulating the expression of amino acid transporters in the small intestine, thereby improving the utilization efficiency of dietary nitrogen [11].

Overall, it is important to provide practical insights into optimizing dietary formulations to enhance the gut health and growth performance of weaned pigs based on protein digestion and fiber fermentation speed. However, few studies have reported the effects of protein digestion and fiber fermentation speeds on the growth performance of weaned pigs. Soybean meal is used as the primary protein source in pig diets, and potato protein meal, wheat protein meal, and cottonseed protein are often chosen to replace soybean meal due to their high protein content and quality. In addition, orange pomace, hawthorn meal, and sugarcane bagasse are common food-processing by-products in China. Orange pomace and hawthorn meal contain many soluble fibers, and sugarcane bagasse contains many insoluble fibers. We hypothesized that dietary protein digestion and fiber fermentation speed would influence growth performance and intestinal health in weaned pigs. Therefore, the aim of this study was to evaluate protein digestion and fiber fermentation patterns among different feed ingredients and explore their effects on growth performance and diarrhea incidence in weaned pigs.

## 2. Materials and Methods

The feeding trial was conducted at the FengNing Swine Research Unit of China Agricultural University (Chengdejiuyun Agricultural and Livestock Co., Ltd., Chengde, China). An in vitro assay was conducted to determine the enzymatic digestion and microbial fermentation patterns of proteins and fibers in different feed ingredients. An in vivo assay was designed to explore the effects of protein digestion and fiber fermentation speeds on growth performance and diarrhea incidence in weaned pigs.

### 2.1. In Vitro Assay

#### 2.1.1. In Vitro Assay of Protein Digestion

The procedures for the in vitro enzymatic digestion assay were as follows:

(1) The four protein ingredients (soybean meal, cottonseed protein, potato protein powder, and corn protein powder) were crushed and sieved through a 0.40 mm mesh, and 0.5 g of the samples was loaded into a digestion tube. Each group included 8 replicates (n = 8);

(2) Buffers were prepared with NaH_2_PO_4_·2H_2_O and Na_2_HPO_4_·2H_2_O, maintaining the pH of the buffer system at 3.5 for the gastric stage and 6.8 for the small intestine stage;

(3) Pepsin (P-7000, >250 U/mg, Sigma-Aldrich, Sigma-Aldrich (Shanghai) Trading Co., Ltd., Shanghai, China), and guar gum (G9310, Solarbio, Beijing Solarbio Science & Technology Co., Ltd., Beijing, China) were dissolved in gastric-stage buffer to obtain an enzyme-containing buffer system. Chloramphenicol (0.5 g) (C8050, Solarbio, Beijing Solarbio Science & Technology Co., Ltd., China) was dissolved in 100 mL of anhydrous ethanol to form a solution, and 0.25 mL was added to a centrifuge tube containing 0.5 g of the sample, followed by 10 mL of enzyme-containing buffer and a few glass beads. In vitro digestion was carried out at 39 °C in a shaking incubator at 280 revolutions per minute;

(4) After 120 min of gastric digestion, 0.048 mol/L NaOH (10 mL) was rapidly added to terminate pepsin digestion. This sample was taken at the 0 min small intestine stage;

(5) Phosphate buffer (pH = 6), trypsin (P7545, 4xUSP, Sigma-Aldrich, Sigma-Aldrich (Shanghai) Trading Co., Ltd., China), starch transglucosidase (A7095, ≥260 U/mL, Sigma-Aldrich, Sigma-Aldrich (Shanghai) Trading Co., Ltd., China), and invertase (I4504, ≥250 U/mL, Sigma-Aldrich, Sigma-Aldrich (Shanghai) Trading Co., Ltd., China) were used to mimic the digestive juices of the small intestinal stage. Next, 5 mL of the mixed enzyme solution was added to the system. The small intestine stage was sampled at 20, 60, 90, 120, 240, 360, and 480 min.

At each sampling point, 0.5 mL of solution was collected from each tube and injected into 5 mL of cold distilled water, cooled for 10 min, centrifuged at 3000× *g* for 10 min, and the supernatant was stored at −20 °C.

The information on the enzymes used in the experiment is shown in Supplementary Table S1.

(6) Calculations

The in vitro digestion kinetics of CP were calculated as follows using the equation proposed in a previous study by Jezierny et al. [12]:

The digestibility at the different in vitro digestion time points (%) = ($\frac{\mathrm{CP}\mathrm{in}\mathrm{the}\mathrm{sample}-\mathrm{CP}\mathrm{in}\mathrm{the}\mathrm{undigested}\mathrm{residues}}{\mathrm{CP}\mathrm{in}\mathrm{the}\mathrm{sample}}$) × 100.

The protein digestibility characteristics of the small intestine stage were calculated using nonlinear regression according to Equation (1). The equation refers to that of Ørskov and McDonald [13], where the maximum value of protein digestibility released over time is called the plateau value (%).

(1) $$D_{t}=D_{0}+P\times (1-e^{-kt})$$

In this equation, *D*_0_ is the protein digestibility (%) in the supernatant at 0 min, and *t* is the digestion time (min). *D_t_* is the protein digestion level (%) at t min. The rate of protein digestion (% per min) is represented by *k*, and *P* is the maximum value of protein digestibility over time, which is called the plateau value (%).

(2) $$V_{0–30}=(C_{30}-C_{0})/30$$

In this equation, *V*_0–30_ indicates the release rate (% per min) within 0 to 30 min of protein digestibility during the small intestinal stage of protein digestion. *C*_30_ and *C*_0_ indicate the protein digestibility (%) at the time points of 30 and 0 min, respectively.

(3) $$V_{30–P}=(C_{P}-C_{30})/240$$

In Equation (3), *V*_30–*P*_ indicates the protein digestibility (% per min) within the in vitro protein slow-digestion phase (30 to 240 min).

#### 2.1.2. In Vitro Microbial Fermentation

The medium for the in vitro microbial fermentation trial was prepared based on the Menke and Steingass Classic Fermentation Medium [14].

(1) Configure the medium

The buffer solution was formulated with 8.32 g/L NaHCO_3_, 0.95 g/L NH_4_HCO_3_, 1.36 g/L Na_2_HPO_4_, 1.47 g/L KH_2_PO_4_, 0.14 g/L MgSO_4_·7H_2_O, 0.30 g/L Na_2_S·9H_2_O, 76.09 mg/L NaOH, 15.69 mg/L CaCl_2_·2H_2_O, 11.89 mg/L MnCl_2_·4H_2_O, 1.19 mg/L CoCl_2_·6H_2_O, 9.51 mg/L FeCl_3_·6H_2_O, and 1.19 mg/L resazurin. The prepared buffer was autoclaved for 15 min at 121 °C and 100 kPa before in vitro microbial fermentation.

(2) Prepare the inoculum

Fresh fecal samples were collected from four healthy piglets with an initial average body weight of 21.50 ± 0.20 kg and an age of 45 ± 3 d to prepare the inoculum. Fecal samples were collected and immediately transferred to an anaerobic workstation. Fresh feces was mixed with 0.9% saline at a ratio of 1:5 (*w*/*v*), and the mixture was filtered through four layers of gauze to obtain a fecal homogenate as the inoculum.

(3) In vitro microbial fermentation

Orange pomace, sugarcane bagasse, and hawthorn powder were added to the fermentation flasks as fermentation substrates. Each group consisted of 8 replicates (n = 8). Finally, 5 mL of inoculum and 25 mL of medium were added to the fermentation flasks and incubated at 39 °C for 48 h. At the end of the in vitro microbial fermentation assay, samples of the fermentation broth were collected to analyze the SCFA concentration and microbial composition.

The microbial fermentability at the end of in vitro microbial fermentation time (%) = ($\frac{\mathrm{TDF}\;\mathrm{in}\;\mathrm{the}\;\mathrm{sample}-\mathrm{TDF}\;\mathrm{in}\;\mathrm{the}\;\mathrm{undigested}\;\mathrm{residues}}{\mathrm{TDF}\;\mathrm{in}\;\mathrm{the}\;\mathrm{sample}}$) × 100.

(4) Gas production kinetics

The change in gas production was recorded using an AGRS-III Microbial Fermentation Gas Production Automatic Recorder (Beijing Boxiang Xingwang Technology Co., Ltd., Beijing, China) for all fermentation flasks. Gas production profiles were fitted to the model as described in a previous report [15].

G=A1+CtB

where G (mL/g) refers to the total substrate gas production, A (mL/g) represents the asymptotic gas production per gram of substrate, B represents the sharpness of the gas production curve, C (h) represents the time required for the actual gas production to reach 1/2 of the theoretical maximum gas production, and t (h) represents the time for gas production to proceed during in vitro microbial fermentation.

#### 2.1.3. Collection and Analysis of the Samples

Dry matter (DM; method 930.15), crude protein (CP; method 984.13), ash (method 942.05), soluble dietary fiber (SDF; method 991.43), insoluble dietary fiber (IDF; method 991.43), and ether extract (EE; method 920.39) were analyzed according to the procedures of the Association of Official Analytical Chemists [16]. Total dietary fiber (TDF) is calculated as the sum of SDF and IDF. Neutral detergent fiber (NDF) and acid detergent fiber (ADF) were analyzed using fiber bags and fiber analyzer equipment (Fiber Analyzer, Ankom Technology, Macedon, NY, USA). Gross energy (GE) was analyzed using an Automatic Isoperibol Oxygen Bomb Calorimeter (Parr 6300 Calorimeter, Parr Instrument Company, Moline, IL, USA). The chemical compositions of potato protein meal, wheat protein meal, soybean meal, cottonseed protein, orange pomace, hawthorn meal, and sugarcane bagasse are shown in Table 1. Samples collected from the bottles at the end of fermentation were centrifuged, and the supernatant was analyzed for SCFA. The fermentation broth was diluted with ultrapure water and filtered using a 0.25 mm Nylon Membrane Filter (Corning Inc., Corning, NY, USA). Acetate, propionate, butyrate, valerate, isobutyrate, and isovalerate were measured using an ion chromatography system (Dionex Corp., Sunnyvale, CA, USA). Total DNA was extracted from the microbial fermentation broth, and representative sequences of amplified sequence variants (ASVs) were obtained after PCR amplification, library construction, and V3–V4 region sequencing of the 16S rRNA gene. An Illumina HiSeq high-throughput sequencing platform was used for this purpose. The PCR reaction, purification and quantification of amplicons, and analysis of raw data were performed according to the methods described in a previous study [17], using UPARSE to cluster operational taxonomic units (OTUs) with 97% similarity. The taxonomy of each 16S rRNA gene sequence was analyzed using the RDP Classifier with a confidence threshold of 90%.

### 2.2. In Vivo Assay

#### 2.2.1. Experimental Design, Animals and Diets

A 2 × 2 factorial treatment arrangement was designed with two different dietary protein digestion speeds and two different fiber fermentation speeds. A total of 192 weaned pigs (Duroc × Landrace × Yorkshire) with an initial average body weight of 6.87 ± 0.14 kg and 24 days of age were selected from the same batch of lactating sows and randomly allotted into four dietary groups: fast-digestion protein with fast-fermentation fiber, fast-digestion protein with slow-fermentation fiber, slow-digestion protein with fast-fermentation fiber, and slow-digestion protein with slow-fermentation fiber. Each dietary treatment included six replicates (pens), with 8 pigs per replicate (4 males and 4 females). All pigs were housed in pens with floors comprising half cement and half woven mesh, and had free access to feed and water throughout the 28 d experiment. The temperature of the pig barn was maintained at 23–28 °C, and the relative humidity was maintained at 60–70%. All diets were formulated following the recommendations of the NRC (2012) to meet the trace mineral and vitamin requirements of weaned pigs [18]. The dietary composition and nutrient levels are presented in Table 2.

#### 2.2.2. Growth Performance and Diarrhea Incidence

All the pigs were weighed on days 0, 14, and 28 after weaning to determine the average daily gain (ADG). Feed intake was estimated by measuring leftovers and waste to calculate the average daily feed intake (ADFI) and feed conversion ratio (FCR; a ratio of ADFI to ADG). The incidence of diarrhea was assessed using fecal scoring, which was performed daily in the morning by the same person. Feces was classified as normal (an absence of diarrhea) or liquid and pasty (the presence of diarrhea). The incidence of diarrhea was calculated using the following equation:

Diarrhea incidence (%) = total number of piglets with diarrhea/(number of piglets × number of experimental days) × 100.

#### 2.2.3. Samples Collection and SCFA Determination

Fresh piglet fecal samples were collected on days 14 and 28 of the experiment, rapidly frozen in liquid nitrogen, and stored at −80 °C for SCFA analysis. The analysis method was the same as that used for the in vitro assay of the measured SCFA.

#### 2.2.4. Statistical Analysis

Data were analyzed using SPSS version 26.0 (IBM Corp., Armonk, NY, USA). All data were checked for normal distribution and homogeneity of variance using Levene’s tests. The indicators related to in vitro protein digestion and the fiber fermentation speeds of different feed ingredients were analyzed using nonlinear models. The interaction effects on growth performance and fecal SCFA concentrations were analyzed using two-way ANOVA, with each replicate as an experimental unit. The statistical model included the fixed main effects of protein digestion speed, fiber fermentation speed, and their interaction effects. Statistical differences were determined using Tukey’s multiple-range test. The results were expressed as least-squares mean and mean standard errors. Statistical significance was set at *p* < 0.05, and a trend towards differences was considered present when 0.05 ≤ *p* < 0.10.

## 3. Results

### 3.1. In Vitro Protein Digestibility

**Figure 1:**
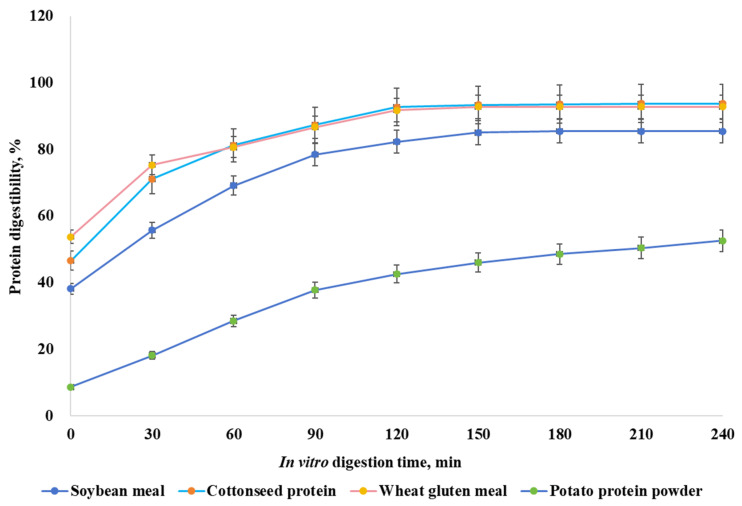
In vitro protein digestibility kinetics of four different feed ingredients.

The in vitro protein digestion kinetics of four different high-protein feed ingredients are shown (Figure 1, Table 3). Wheat gluten meal, soybean meal, and cottonseed protein exhibited rapid digestion, achieving >80% digestibility within 60 min, whereas potato protein powder did not (*p* < 0.05). Wheat gluten meal displayed the fastest initial digestion speed, with 60% protein digestibility within the first 20 min. However, the protein digestibility of potato protein powder remained below 40% until 90 min.

### 3.2. In Vitro Microbial Fermentation of Fiber Components

**Figure 2:**
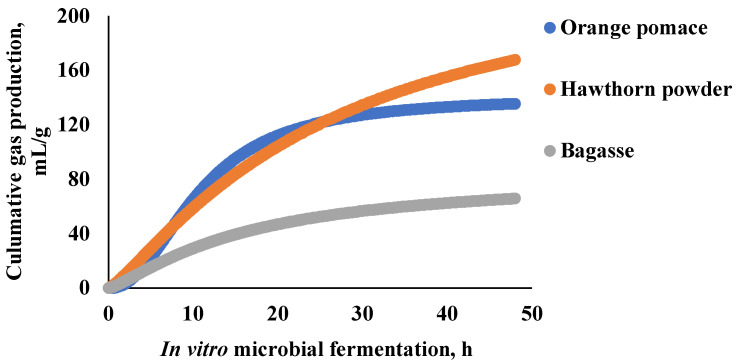
In vitro fermentation gas production kinetics of different feed ingredients.

**Figure 3:**
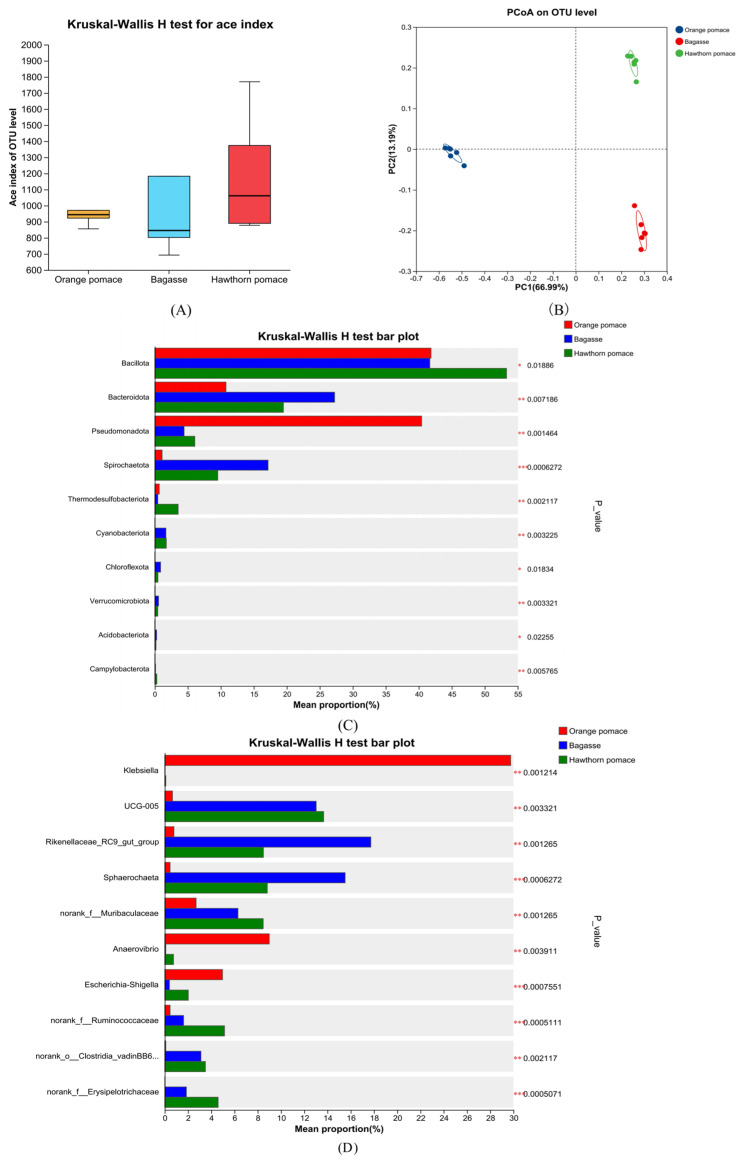
The microbial conditions of the fermentation broth at the end of fermentation. (**A**) An analysis of the alpha diversity ace index. (**B**) The beta diversity index. (**C**) Differential microbes at the phylum level. (**D**) Differential microbes at the genus level: the red bar represents orange pomace, blue represents bagasse, and green represents hawthorn powder. * *p* < 0.05, ** *p* < 0.01, and *** *p* < 0.001.

The in vitro microbial fermentation of three different high-fiber feed ingredients is shown (Figure 2). The gas production kinetics of orange pomace, sugarcane bagasse, and hawthorn powder were as follows: gas production (mL/g) = 140.66/[1 + (10.67/t)^2.18^], gas production (mL/g) = 140.66/[1 + (15.80/t)^1.27^], and gas production (mL/g) = 245.96/[1 + (25.70/t)^1.22^], respectively (Table 4). Hawthorn powder and orange pomace exhibited faster gas production than sugarcane bagasse (*p* < 0.05). In addition, hawthorn powder showed greater in vitro microbial fermentability of fiber components than orange pomace and sugarcane bagasse (*p* < 0.05). The SCFA concentration and proportion from the in vitro microbial fermentation of three different high-fiber feed ingredients are shown (Table 5). Orange pomace yielded the highest acetate concentration, which was significantly higher than that of hawthorn powder (*p* < 0.05) and sugarcane bagasse (*p* < 0.05). Propionate and butyrate concentrations followed a similar trend, with orange pomace producing the highest amounts. The valerate concentration did not differ significantly among the groups (*p* > 0.05). In addition, there were no significant differences in the proportions of acetate, propionate, and butyrate accounted for total SCFA among orange pomace, hawthorn powder, and sugarcane bagasse (*p* > 0.05). The microbial composition analysis of the fermentation broth at the end of in vitro fermentation is shown in Figure 3. No significant differences were observed in the microbial alpha and beta diversities among the different dietary groups (*p* > 0.05). However, hawthorn powder significantly enriched the *Bacillus phylum* compared to orange pomace and sugarcane bagasse (*p* < 0.05). Orange pomace showed a significant increase in *Pseudomonas phylum* and a decrease in *Bacteroidota phylum* abundance compared with hawthorn powder and sugarcane bagasse (*p* < 0.05). At the genus level, orange pomace fermentation broth significantly enriched *Klebsiella*, *Escherichia–Shigella*, and *Anaerovibrio genera* (*p* < 0.05). Compared with orange pomace, both sugarcane bagasse and hawthorn meal groups showed enrichment of UCG-005, *Rikenellaceae_RC9_gut_group,* and *Sphaerochaeta* (*p* < 0.05).

### 3.3. Effects of Protein Digestion and Fiber Fermentation Speeds on Growth Performance and Diarrhea Incidence of Weaned Pigs

The effects of different protein–fiber combinations on the growth performance of weaned piglets are summarized in Table 6. No significant interactions between protein digestion and fiber fermentation rates were observed on growth performance (*p* > 0.05). On day 28, the piglets fed slow-digestion protein + fast-fermentation fiber exhibited a numerically higher final body weight (17.85 kg) than those in the other groups, although no significant differences in growth performance were observed among the dietary treatments (*p* > 0.05). Similarly to ADFI and ADG, the slow-digestion protein + fast-fermentation fiber group showed the highest ADFI (639 g/d) and ADG (395 g/d), while the fast-digestion protein + slow-fermentation fiber group had the lowest values (596 g/d and 363 g/d, respectively). No significant interactions in the diarrhea incidence of weaned pigs between protein digestion and fiber fermentation speeds were observed (*p* > 0.05). However, fast-fermentation fiber speed significantly influenced the incidence of diarrhea. Piglets fed slow-fermentation fiber exhibited a lower incidence of diarrhea than those fed fast-fermentation fiber (*p* < 0.05). Notably, the fast-digestion protein + slow-fermentation fiber combination resulted in the lowest incidence of diarrhea.

### 3.4. Effects of Protein Digestion and Fiber Fermentation Speeds on Fecal SCFA Concentrations

No significant interactions between protein digestion and fiber fermentation speed were observed on fecal SCFA concentration (Table 7; *p* > 0.05). However, the rates of protein digestion and fiber fermentation can affect fecal SCFA concentrations in weaning piglets. Slow-digestion protein significantly increased the concentrations of valerate and branched-chain SCFAs compared with fast-digestion protein (*p* < 0.05), and fast-fermentation fiber significantly increased acetate concentration compared with slow-fermentation fiber (*p* < 0.05).

## 4. Discussion

### 4.1. In Vitro Protein Digestion and Fiber Fermentation Properties

In the current study, wheat gluten meal demonstrated the fastest digestion speed (up to 60% within 20 min), which may be attributed to its unique gliadin–gluten matrix. Wheat gliadins are monomeric proteins that are more readily hydrolyzed by pepsin than glutenins, which have a polymeric structure [19]. This observation is consistent with previous findings that 85–90% of wheat proteins are digested in the porcine ileum due to their low trypsin inhibitor content and high solubility at gastric pH [20]. In addition, our study showed the digestibility of cottonseed protein to be up to 80% within 60 min, which is consistent with Zhuo et al. [21], who reported that the ileal digestibility of CP was approximately 82% in growing pigs. In addition, the high digestibility of cottonseed protein may be caused by the reduced anti-nutritional factors (e.g., trypsin inhibitors) compared with soybean meal, which showed slower protein digestion kinetics [22].

The microbial fermentation patterns of fiber reflect differences in cell-wall structure and soluble fiber fractions. The rapid fermentation speed of orange pomace is associated with its high pectin content, which is readily metabolized by pectinolytic bacteria [23]. The high acetate levels produced by orange pomace are consistent with the pectin-specific fermentation pathway [24]. Hawthorn powder has higher gas and acetic acid yields, which may be related to its high soluble hemicellulose content [25]. Alternatively, the soluble/insoluble fiber ratio may be balanced, and microorganisms can gradually access the hemicellulose fraction while maintaining prolonged fermentation. The slower microbial fermentation of sugarcane bagasse is caused by its lignocellulosic matrix, which acts as a physical barrier to microbial enzymes [26].

### 4.2. In Vitro Microbial Composition

The in vitro microbial fermentation of orange pomace led to an increase in the relative abundance of *Klebsiella* and *Escherichia–Shigella* associated with rapid microbial degradation, which may create transient acidic conditions in the proximal colon (pH < 5.5) favorable to acid-resistant opportunistic pathogens. Although rapidly fermenting fibers such as pectin have traditionally been considered to exert beneficial effects on the host due to their ability to produce large amounts of SCFA, their rapid depletion may lead to substrate scarcity in the distal colon, which may trigger the compensatory fermentation of proteins and proliferation of proteolytic bacteria (e.g., *Escherichia–Shigella*) [27]. This phenomenon is consistent with several studies that suggest that abrupt shifts in nutrient availability can destabilize microbial ecosystems and increase susceptibility to dysbiosis [28].

In contrast, sugarcane bagasse, which has slow microbial fermentation properties, supports a more stable microbial environment characterized by an abundance of fiber-degrading *Rikenellaceae_RC9_gut_group* and mucin-associated *norank_f__Muribaculaceae* microbes. These taxa are indispensable for maintaining intestinal barrier function: members of the *Rikenellaceae* family metabolize complex polysaccharides to butyrate, a key energy source for colonocytes, while the Muribaculaceae enhance the integrity of the mucus layer by regulating thrush cell activity [29]. The significantly lower abundance of Escherichia coli in the sugarcane bagasse and hawthorn meal groups could explain the lower rate of diarrhea incidence in the slow fermentation groups, possibly due to the competitive rejection of beneficial taxa and the sustained SCFA production that maintains the colonic pH above 6.0, an unfavorable environment for intestinal pathogens to thrive [30]. Hawthorn powder, with its moderate fermentation speed, uniquely enriched UCG-005 (a *Clostridiales genus*) and *Anaerovibrio*, a propionate-producing bacterium. This aligns with its high total SCFA yield in vitro, particularly propionate, which exerts anti-inflammatory effects via GPR41/43 signaling and enhances hepatic gluconeogenesis [31].

### 4.3. Growth Performance

The study revealed no significant interactive effects between protein digestion and fiber fermentation rates on the growth performance and intestinal health of weaned piglets. However, their individual effects on protein digestion and fiber fermentation dynamics were observed. The in vitro and in vivo results collectively provide mechanistic insights into how dietary components influence physiological outcomes.

On the one hand, the in vitro protein digestion profiles aligned with in vivo observations. Surprisingly, protein digestion rates had no significant impact on growth performance (Table 6) despite the rapid in vitro digestion kinetics of cottonseed and wheat proteins, which is hypothesized to optimize nitrogen utilization in the foregut and enhance nutrient absorption [20]. For instance, previous studies have reported that rapidly digestible casein combined with pectin improves the average daily gain (ADG) of weaned pigs [32]. However, similar to this study, other findings suggest that the advantages of rapidly digestible proteins are context-dependent, emerging only under acute stress or in diets already characterized by high digestibility [33].

Conversely, in vitro digestion kinetics revealed that potato protein exhibited significantly slower proteolysis (40% digestion within 90 min), leading to a greater amount of residual protein entering the hindgut. This aligns with the in vivo observations of higher fecal valerate and branched-chain fatty acid (BCFA) concentrations in pigs fed slow-digesting protein sources (Table 6 and Table 7). BCFAs are microbial metabolites derived from the fermentation of branched-chain amino acids (BCAAs), such as valine, leucine, and isoleucine. The delayed digestion of potato protein likely prolongs the availability of these amino acids in the colon, where commensal microbes (e.g., Clostridium and Bacteroides) metabolize them into BCFAs via deamination and decarboxylation pathways. Moderate BCFA levels may enhance intestinal barrier function by upregulating tight-junction proteins [34]. However, excessive BCFA concentrations could compromise epithelial integrity, which may explain why, during days 0–14, the difference in microbial metabolites of branched-chain fatty acids between the slow-digestion protein group and the fast-digestion group was not significant, with no marked difference in diarrhea rate. Compared with the fast-digestion group, from days 14 to 28, as the concentration of branched-chain fatty acids increased in the slow-digestion group, the diarrhea rate also increased (Table 6 and Table 7).

In contrast, in vitro fiber fermentation dynamics (gas production and SCFA profiles) directly translate to in vivo physiological effects. Fiber fermentation rates had a more pronounced impact on growth performance and intestinal health. Orange peel residue, characterized by rapid fermentation kinetics, generated high acetate concentrations and elevated total SCFA production in vitro, significantly higher than that of sugarcane bagasse (19.94 vs. 14.04 mmol/L). As acetate serves as the primary energy source for colonocytes, rapidly fermentable fibers like orange peel residue may enhance intestinal epithelial energy metabolism, sparing dietary amino acids from catabolism and improving protein utilization efficiency [35]. Consequently, pigs fed orange peel residue exhibited a trend toward higher ADG, accompanied by increased total SCFA (36.91 vs. 35.41 mmol/L, *p* = 0.045) and acetate levels (18.13 vs. 13.87 mmol/L, *p* = 0.034) compared to fast-fermenting fibers after 28 d. However, the in vitro enrichment of *Klebsiella* and *Escherichia–Shigella* in the orange peel residue groups may explain the elevated diarrhea risk observed in vivo, as these taxa thrive under transient acidic conditions and may destabilize the microbial equilibrium.

In contrast, sugarcane bagasse, with its slow in vitro fermentation kinetics, was associated with a reduced incidence of diarrhea. This is mechanistically linked to its lignocellulosic structure, which prolongs fermentation duration, enhances intestinal motility, increases fecal bulk, and accelerates evacuation, thereby minimizing harmful metabolite retention [26]. Furthermore, slow-fermenting fibers stabilize colonic pH, suppressing pathogenic proliferation and favoring beneficial taxa. Consistent with the in vitro findings, sugarcane bagasse reduced the abundance of proteolytic bacteria (e.g., *Escherichia*) and promoted fiber-degrading microbes, such as *Rikenellaceae_RC9_gut_group* and *norank_f__Muribaculaceae*, both of which are critical for maintaining gut health. *Rikenellaceae_RC9_gut_*group metabolizes complex polysaccharides into butyrate, which enhances epithelial barrier function via occludin upregulation. *Norank_f__Muribaculaceae* regulates mucus-layer integrity by stimulating goblet-cell activity, which physically blocks pathogen adhesion. This explains the 38% reduction in the incidence of diarrhea (8.05% vs. 12.69%, *p* = 0.011). These observations corroborate prior studies demonstrating that slow-fermenting fibers stabilize the gut microbiota and mitigate the risk of pathogenic overgrowth [30]. In addition, the results of in vitro fermentation for microbial composition are presented. Additionally, sugarcane bagasse increased the fecal concentrations of butyrate and valerate, which are SCFAs associated with anti-inflammatory effects and improved intestinal barrier integrity.

## 5. Conclusions

There were no significant differences in the interactions between protein digestion and fiber fermentation speeds on growth performance and fecal SCFA concentrations in weaned pigs. However, the diet supplemented with fast-fermenting fiber showed a tendency to improve growth performance, while slow-fermenting fiber appeared to be more effective in reducing the incidence of diarrhea in weaned pigs. In addition, the diet supplemented with slow-digesting protein did not influence growth performance but increased the production of valerate and BCFA, indicating that slow-digesting protein increases the microbial fermentation of dietary protein. Overall, our findings suggest that pig farmers should choose fast- and slow-fermenting fibers to formulate pig feed based on the actual increase in the growth rate of piglets and the reduction in the incidence of diarrhea in weaned pigs. However, the requirements for dietary nutrients with different digestion and fermentation rates, and how they pertain to pigs, need to be further explored.

## Figures and Tables

**Table 1:** Chemical composition of feed ingredients used in the study (%, as-fed basis).

Items	Soybean Meal	Cottonseed Protein	Wheat Protein Powder	Potato Protein Powder	Orange Pomace	Sugarcane Bagasse	Hawthorn Powder
GE, MJ/kg	17.26	18.61	21.73	20.65	16.23	15.85	15.98
DM	89.23	89.68	92.63	93.26	91.42	90.52	90.45
CP	43.69	56.42	67.86	78.45	5.71	2.59	3.85
TDF	19.04	8.03	7.85	3.65	43.7	91.5	67.3
SDF	2.41	1.36	1.04	0.45	10.9	12.4	2.1
IDF	16.63	6.67	6.81	3.2	32.8	79.1	65.2
NDF	12.44	7.85	6.13	3.02	28.8	75.9	66.43
ADF	5.82	2.12	2.89	2.55	18.5	50.7	42.65
EE	1.12	0.45	1.42	2.53	0.91	0.65	0.53
Ash	5.23	2.99	1.97	1.28	5.94	4.52	4.57

The chemical compositions of the samples were analyzed in duplicate. GE, gross energy; DM, dry matter; CP, crude protein; TDF, total dietary fiber; SDF, soluble dietary fiber; IDF, insoluble dietary fiber; NDF, neutral detergent fiber; ADF, acid detergent fiber; Ash, crude ash; EE, ether extract.

**Table 2:** Dietary composition and nutrient levels (%, as-fed basis).

Items	Dietary Treatments			
	Fast Protein + Fast Fiber	Fast Protein + Slow Fiber	Slow Protein + Fast Fiber	Slow Protein + Slow Fiber
Corn	60.50	60.50	59.89	59.89
Soybean meal			7.50	7.50
Cottonseed protein	6.50	6.50		
Potato protein powder			5.00	5.00
Wheat protein powder	5.00	5.00		
Orange pomace	5.00		5.00	
Sugarcane bagasse		5.00		5.00
Soy protein concentrate	5.00	5.00	5.00	5.00
Extruded full-fat soybean	6.00	6.00	6.00	6.00
Whey powder, 3.8%	5.00	5.00	5.00	5.00
Soy oil	2.50	2.50	2.50	2.50
Dicalcium phosphate	1.40	1.40	1.40	1.40
Limestone	1.00	1.00	1.00	1.00
NaCl	0.30	0.30	0.30	0.30
L-Lysine HCl	0.84	0.84	0.57	0.57
DL-Methionine	0.14	0.14	0.11	0.11
L-Threonine	0.27	0.27	0.18	0.18
L-Tryptophan	0.05	0.05	0.05	0.05
Premix ^(1)^	0.50	0.50	0.50	0.50
Total	100.00	100.00	100.00	100.00
Nutrient levels, % ^(2)^				
Gross energy, MJ/kg	16.85	16.73	16.77	16.62
Crude protein	19.11	19.27	19.51	19.43
Total dietary fiber	9.35	11.32	9.56	11.14
Soluble dietary fiber	1.03	1.12	0.93	1.22
Insoluble dietary fiber	8.32	10.20	8.63	9.92
Neutral detergent fiber	9.21	10.88	9.37	10.59
Acid detergent fiber	4.24	5.12	4.73	4.94
Ether extract	4.63	4.89	4.44	4.71

^(1)^ Premix provides a per kg complete diet: Vitamin A, 12,000 IU; Vitamin D_3_, 2000 IU; Vitamin E, 30 IU; Vitamin K_3_, 3.0 mg; Vitamin B_6_, 3.0 mg; Vitamin B_12_, 12 μg; Riboflavin, 4.0 mg; Thiamine, 1.5 mg; Niacin, 40 mg; Pantothenic acid, 15 mg; Folic acid, 0.7 mg; Biotin, 44 μg; choline chloride, 400 mg; copper, 10 mg; iron, 90 mg; zinc, 80 mg; manganese, 30 mg; iodine, 0.35mg; selenium, 0.3 mg. ^(2)^ The analyzed values are shown, and the data were analyzed in duplicate.

**Table 3:** Kinetic parameters of in vitro protein digestion from different types of protein feed ingredients.

Items ^(1)^	Soybean Meal	Cottonseed Protein	Wheat Gluten Meal	Potato Protein Powder	SEM	*p*-Value ^(2)^
*P* (%/g)	85.45 ^b^	93.73 ^a^	92.75 ^a^	52.56 ^c^	2.531	0.032
*V*_0–30_ (%/min)	0.586 ^ab^	0.818 ^a^	0.719 ^a^	0.316 ^b^	0.044	0.045
*V*_30–*P*_ (%/min)	0.198	0.15	0.116	0.115	0.023	0.450
*k*	0.032	0.028	0.035	0.019	0.002	0.092

^(1)^ The rate of protein digestion (% per min) is represented by *k*, and *P* is the maximum value of protein digestibility over time, which is the plateau value (%). *V*_0–30_ indicates the release rate (% per min) within 0 to 30 min of protein digestibility during the small intestinal stage. *V*_30–*P*_ indicates the protein digestibility (% per min) within the in vitro protein slow-digestion phase (30 to 240 min). ^(2)^ Different letters in the same row represent significant differences (*p* < 0.05). SEM, standard error of the mean.

**Table 4:** Kinetic parameters of gas production.

Items ^(1)^	Orange Pomace	Sugarcane Bagasse	Hawthorn Powder	SEM	*p*-Value ^(2)^
A, mL	140.66 ^b^	81.82 ^c^	245.96 ^a^	10.27	0.01
B	2.18 ^a^	1.27 ^b^	1.22 ^b^	0.17	0.01
C, h	10.67 ^c^	15.80 ^b^	25.70 ^a^	1.25	0.01

^(1)^ A (mL/g) represents the asymptotic gas production per gram of substrate, B represents the sharpness of the gas production curve, and C (h) represents the time required for the actual gas production to reach 1/2 of the theoretical maximum gas production. ^(2)^ Different letters in the same row represent significant differences (*p* < 0.05). SEM, standard error of the mean.

**Table 5:** Concentration and proportion of SCFA at the end of in vitro fermentation.

Items ^(1)^	Orange Pomace	Hawthorn Powder	Sugarcane Bagasse	SEM	*p*-Value
In vitro microbial fermentation					
Fermentability, %	82.65 ^a^	37.16 ^b^	46.85 ^b^	3.65	0.012
Concentration, mmol/L					
Acetate	19.94 ^a^	14.04 ^ab^	10.46 ^b^	1.67	0.016
Propionate	2.87	2.04	1.87	0.43	0.138
Butyrate	2.05	1.57	1.10	0.39	0.221
Valerate	0.51	0.39	0.27	0.24	0.442
Total SCFA	25.38 ^a^	18.05 ^ab^	13.70 ^b^	1.92	0.011
Proportion, %					
Acetate	78.57	77.81	76.37	3.53	0.835
Propionate	11.31	11.30	13.62	1.25	0.429
Butyrate	8.09	8.72	8.00	0.84	0.957
Valerate	2.02	2.18	2.00	0.23	0.883

^(1)^ Different letters in the same row represent significant differences (*p* < 0.05). SEM, standard error of the mean.

**Table 6:** Effects of different protein digestion and fiber fermentation speeds on growth performance of weaned pigs.

Items ^(1)^	Protein Digestion Speed		Fiber Fermentation Speed		SEM	*p*-Value ^(2)^		
	Fast Protein	Slow Protein	Fast Fiber	Slow Fiber		Protein Digestion Speed	Fiber Fermentation Speed	Interaction
Body weight, kg								
d 0 after weaning	6.80	6.80	6.80	6.80	0.18	0.995	0.993	0.982
d 14 after weaning	11.19	11.33	11.42	11.11	0.26	0.755	0.444	0.684
d 28 after weaning	17.25	17.50	17.69	17.06	0.42	0.524	0.187	0.795
ADFI, g/d								
d 0–14	477	491	500	468	13.85	0.315	0.086	0.423
d 14–28	735	749	755	729	17.32	0.365	0.168	0.637
d 0–28	606	620	628	599	15.26	0.337	0.122	0.568
ADG, g/d								
d 0–14	314	324	330	308	8.13	0.533	0.063	0.624
d 14–28	433	441	448	425	9.97	0.732	0.093	0.473
d 0–28	373	383	389	367	9.08	0.662	0.087	0.526
FCR								
d 0–14	1.52	1.52	1.52	1.52	0.01	0.821	0.726	0.425
d 14–28	1.70	1.70	1.69	1.72	0.02	0.763	0.633	0.591
d 0–28	1.63	1.62	1.62	1.63	0.02	0.795	0.752	0.535
Diarrhea incidence, %								
d 0–14	8.74	8.09	10.64 ^a^	6.20 ^b^	1.52	0.685	0.015	0.488
d 14–28	11.46	13.18	14.75 ^a^	9.90 ^b^	1.71	0.449	0.033	0.523
d 0–28	10.10	10.64	12.69 ^a^	8.05 ^b^	1.66	0.621	0.011	0.515

^(1)^ ADFI, average daily feed intake; ADG, average daily gain; BW, body weight; FCR, feed conversion ratio; SEM, standard error of the mean. ^(2)^ Different letters in the same row represent significant differences (*p* < 0.05).

**Table 7:** Effects of different protein digestion and fiber fermentation speeds on fecal SCFA concentrations in weaned pigs.

Items ^(1)^	Protein Digestion Speed		Fiber Fermentation Speed		SEM	*p*-Value ^(2)^		
	Fast Protein	Slow Protein	Fast Fiber	Slow Fiber		Protein Digestion Speed	Fiber Fermentation Speed	Interaction
d 14								
Acetate	14.11	13.48	15.05 ^b^	12.53 ^a^	1.02	0.832	0.045	0.651
Propionate	6.45	6.50	6.58	6.37	0.56	0.943	0.832	0.871
Butyrate	4.06	3.72	4.15	3.64	0.41	0.425	0.917	0.768
Valerate	0.95	0.70	0.85	0.80	0.16	0.575	0.934	0.424
Branched SCFAs	1.92	1.96	1.84	2.04	0.69	0.891	0.819	0.425
Total SCFAs	27.48	26.35	28.46	25.37	1.67	0.628	0.179	0.773
d 28								
Acetate	15.83	16.17	18.13 ^a^	13.87 ^b^	1.35	0.878	0.034	0.731
Propionate	8.33 ^b^	10.96 ^a^	9.21	10.07	0.82	0.043	0.563	0.621
Butyrate	5.16	6.73	5.61	6.28	0.57	0.073	0.283	0.826
Valerate	0.95 ^b^	1.94 ^a^	1.29 ^b^	1.60 ^a^	0.18	0.022	0.013	0.338
Branched SCFAs	2.21 ^b^	3.68 ^a^	2.68	3.20	0.43	0.017	0.425	0.852
Total SCFAs	32.46	39.47	36.91	35.01	1.89	0.065	0.448	0.769

^(1)^ SCFAs, short-chain fatty acids; SEM, standard error of the mean. ^(2)^ Different letters in the same row represent significant differences (*p* < 0.05).

## Data Availability

The datasets used in this study are available from the corresponding author upon reasonable request.

## Supplementary Materials

The following supporting information can be downloaded at: https://www.mdpi.com/article/10.3390/ani15111669/s1. Table S1: Information on enzymes used in the in vitro digestion assay.
