# Supplementary material for: Effects of Dietary Fiber Fermentation and Protein Digestion Properties on Growth Performance and Microbial Metabolites in Weaned Pigs
Source: Animals (Basel). 2025 Jun 5;15(11):1669. doi: 10.3390/ani15111669 (PMC12153613; doi:10.3390/ani15111669)
Supplement: Supplementary file 1 [file animals-15-01669-s001.zip › animals-3632516-supplementary.pdf]

Supplementary Table S1: Information on enzymes used in the in vitro digestion assay

**Enzyme/Reagent**	**Product Code**	**Activity/Specification**	**Supplier**	**Address**
Pepsin	P-7000	>250 U/mg	Sigma-Aldrich	Shanghai, China
Trypsin	P7545	4×USP	Sigma-Aldrich	Shanghai, China
Starch Transglucosidase	A7095	≥260 U/mL	Sigma-Aldrich	Shanghai, China
Invertase	I4504	≥250 U/mL	Sigma-Aldrich	Shanghai, China
